# Prehypertension and Chronic Kidney Disease in Chinese Population: Four-Year Follow-Up Study

**DOI:** 10.1371/journal.pone.0144438

**Published:** 2015-12-15

**Authors:** Hao Xue, Jianli Wang, Jinhong Hou, Junjuan Li, Jingsheng Gao, Shuohua Chen, Hang Zhu, Shouling Wu

**Affiliations:** 1 Department of Cardiology, Chinese People’s Liberation Army General Hospital, 28 Fuxing Road, Beijing 100853, People’s Republic of China; 2 Department of Cardiology, Kailuan Hospital, Hebei United University, Tangshan, China; The University of Manchester, UNITED KINGDOM

## Abstract

Hypertension is a well established cause of chronic kidney disease (CKD). However, the effect of prehypertension on risk of CKD is controversial. The aim of this study is to determine whether prehypertension increases the risk of CKD events in the Chinese population. We enrolled 20,034 with prehypertension and 12,351 with ideal blood pressure in this prospective study. CKD was defined as an estimated glomerular filtration rate (eGFR) <60 ml/min 1.73m^2^. The new occurrences of CKD events were collected during follow-up. Cumulative survival and freedom for the occurrence of new CKD events was analyzed using the Kaplan-Meier approach. Multivariate Cox Regression was used to analyze the effect of prehypertension on CKD. The median follow-up time was 47 (interquartile range 44–51) months. 601 new onset CKD events occurred during the follow-up period. The cumulative incidence of new CKD events was higher in the prehypertensive population than that in the ideal blood pressure population (2.10% vs 1.46%, P = 0.0001). Multivariate Cox Regression showed that relative risks (RRs) for the new onset CKD events in the prehypertensive population were 1.69 (95% confidence intervals (CI): 1.41~2.04, P = 0.001) higher than those in the ideal blood pressure population. Similarly, the risks were 1.68 (95% CI: 1.33~2.13 P = 0.001) times higher in females and 2.14 (95% CI: 1.58~2.91 P = 0.001) times higher in males by adjustment for traditional CV risk factors. Our findings demonstrated prehypertension is an independent risk factor for the occurrence of new CKD events in the Chinese population.

## Introduction

Chronic kidney disease (CKD) has become an important public health problem in China. According to recent epidemiological data, the prevalence of CKD in China has reached 10.8%, which means that approximately 119.5 million people currently suffer from CKD in China[1]. Hypertension is one of the leading causes of CKD[2, 3]. Previous study showed that about 25.3% of hypertensive patients had some degree of CKD in the Turkey-CREDIT Studys[4]. Therefore, the rapid increase of prevalence of hypertension might be predicted to drive epidemics of CKD in China.

In 2003,“prehypertension” is defined in the Seventh Report of the Joint National Committee on Prevention, Detection, Evaluation, and Treatment of High Blood Pressure (JNC7), which is associated with a risk of developing hypertension[5, 6]. Epidemiologic study shows that the prevalence of prehypertension was 56.9% in China[7]. About 25.2, and 32.4% normal, and high-normal BP was developing hypertension[8]. Moreover, prehypertension is related to morbidity and mortality of cardiovascular disease[9, 10]. The kidney is also a critical target organ of hypertension-related damage. The level of blood pressure is associated with CKD and end-stage renal disease (ESRD), and lowering of blood pressure can delay the deterioration of renal function in hypertensive patients[6, 11, 12]. Similarly, prehypertension ought to be associated with CKD. However, up to now, the effect of prehypertension on the risk of CKD has still been controversial. Therefore, in order to address this issue, we prospectively investigate the potential relationship of prehypertension with CKD in the Kailuan cohort study (ChiCTR-TNC-11001489).

## Materials and Methods

### Study design

A total of 101,510 working and retired employees of Tangshan Kailuan company were recruited from the Kailuan General Hospital and its 10 affiliated hospitals (including Kailuan Lindsey Hospital, Kailuan Zhaogezhuang Hospital, Kailuan Tang Village Hospital, Kailuan Fangezhuang Hospital, Kailuan Lujiatuo Hospital, Kailuan Jinggezhuang Hospital, Kailuan Linnancang Hospital, Kailuan Qianjiaying Hospital, Kailuan Majiagou Hospital, and Kailuan Hospital Branch) to take medical examinations from July 2006 to October 2007. Among them were 57838 subjects comprised the BP and kidney disease Cohort Studies. According to The Seventh Report of the Joint National Committee on Prevention, Detection, Evaluation, and Treatment of High Blood Pressure (JNC7) criteria[6]: Prehypertension was defined as systolic blood pressure (SBP) at 120–139 mmHg or diastolic blood pressure (DBP) at 80–89 mmHg, the ideal blood pressure was defined as BP levels below 120/80 mmHg. Subjects with the following criteria were excluded: (1) incomplete original clinical data related to the study. (2) According to the JNC7 criteria, hypertension was defined as: SBP ≥140 mmHg and/or DBP≥90mmHg mmHg on average of two measurements or by current antihypertensive treatment. (3) eGFR<60 ml/min 1.73m^2^. (4) Infectious diseases, cancer, blood diseases, severe liver diseases, heart dysfunction, or autoimmune diseases. (5) History of primary renal diseases or renal artery stenosis.

Based on these criteria, 32,385 subjects (20,034 met the JNC 7 prehypertension diagnostic criteria, 12,351 met the JNC 7 diagnostic criteria for ideal blood pressure) were included in the present study, for which all the clinical data met for the present analysis.

All participants were Han nationality. The study was approved by the Ethics Committee of the Kailuan General Hospital. The study was performed according to the guidelines of Helsinki Declaration. The informed consent forms were written by all participants.

### Data collection

Questionnaires were administered face to face by research doctors investigators as previously described[13, 14]. Medical information was recorded from all subjects, including age, gender, family history of hypertension, diabetes mellitus, coronary heart disease and stroke, medical history, alcohol intake, cigarette smoking, dietary intake data, physical activity.

Height and weight were measured. Height was measured to the nearest 0.1 cm using a tape rule, and weight was measured to the nearest 0.1 kg using calibrated platform scales. Body mass index (BMI) was calculated by using the formula of weight (kg)/height (m^2^).

### Blood pressure measurements

Blood pressure was measured by professional doctors with a standardized mercury sphygmomanometer after at least 5 min rest at the subject’s left upper arm in the sitting position. Two readings each of SBP and DBP were recorded at a 5-min interval. The average of the two readings was used for analysis.

### Biochemical variables determination

Blood samples were collected in tubes containing EDTA after an overnight fasting, and were centrifuged at 3000 g for 10 min (centrifuge radius of 17 cm) at room temperature. All samples were measured within 4 hours. Fasting blood glucose (FBG) was measured by hexokinase/glucose-6-phosphate dehydrogenase method. Total cholesterol (TC) and triglyceride (TG), high-density lipoprotein cholesterol (HDL-C), low-density lipoprotein cholesterol (LDL-C) and serum creatinine (Scr) were determined enzymatically (inter-assay coefficient of variation <10%; Mind Bioengineering Co. Ltd, Shanghai, China) with an automatic biochemical analyzer (Hitachi 747; Hitachi, Tokyo, Japan) at the central laboratory of the Kailuan General Hospital.

### Follow-up and new CKD events

We followed all participants from the first physical examination from June 2006 and October 2007 to December 31, 2010. The estimated glomerular filtration rate (eGFR) is used to assess kidney function by using measured Scr. CKD was defined as an eGFR of less than 60 mL/min per 1.73 m^2^ occurring at any time during the follow-up period[15]. The primary outcome was defined as the occurrence of CKD at the time of medical examination during follow-up. The eGFR was calculated by using the Chronic Kidney Disease Epidemiology Collaboration (CKD-EPI) equation[16]:

$$\text{eGFR }\left(\text{mL/min/1}{\text{.73m}}^{\text{2}}\right)=144\times \left(\text{Scr/0.7}\right)-0.329\times \left(0.993\right)\text{Age(if female Scr}\leq \text{62umol/L)}$$

$$\text{eGFR}\left(\text{mL/min/1}{\text{.73m}}^{\text{2}}\right)=144\times \left(\text{Scr/0.7}\right)-1.209\times \left(0.993\right)\text{Age }\left(\text{if female Scr }>\text{62umol/L}\right)$$

$$\text{eGFR}\left(\text{mL/min/1}{\text{.73m}}^{\text{2}}\right)=141\times \;\left(\text{Scr/0.9}\right)-0.411\times \left(0.993\right)\text{Age(if male Scr}\leq \text{80umol/L)}$$

$$\text{eGFR}\left(\text{mL/min/1}{\text{.73m}}^{\text{2}}\right)=141\times \;\left(\text{Scr/0.9}\right)-1.209\times \left(0.993\right)\text{Age}\left(\text{if male Scr }>\text{80umol/L}\right)$$

### Statistical analysis

All statistical analyses were performed using the SPSS13.0 software package (SPPS Inc., Chicago, IL, USA). Continuous variables were described as mean±standard deviation (SD). All categorical variables were presented as numbers and percentages. The comparisons in baseline characteristics between the prehypertension group and ideal blood pressure group were analyzed with chi-square for categorical variables and using Student’s t tests for continuous variables.

The cumulative incidence of new CKD events was calculated using the Kaplan-Meier approach, and the difference in cumulative hazard risks between the two groups was compared with a log-rank test. Multivariate Cox regression was used to analyse the effect of prehypertension on new CKD events at follow-up, adjusting for age, sex, smoking, drinking, history of diabetes mellitus, hyperlipidemia, BMI, TC, LDL-C, TG, and FBG. The hazard ratios (HR) and 95% confidence intervals (CI) of the incidence of the new CKD events in relation to prehypertension were also estimated from Cox proportional hazard models. Model 1 was analyzed without adjusting risk factors. Model 2 was performed adjusting for age and sex. Finally Model 3 was further adjusted for smoking, drinking, diabetes mellitus, hyperlipidemia, BMI, TC, LDL-C, TG, and FBG based on the variables in Model 2. p<0.05 (two sided) was considered statistically significant.

## Results

### Baseline clinical characteristics

Our study included 20,034 participants in the prehypertension group and 12,351 in the ideal blood pressure group in the final statistical analysis. Baseline clinical characteristics between the prehypertension group and the ideal blood pressure group compared are shown in Table 1. Subjects with prehypertension tended to be older and heavier than those with the ideal blood pressure. In the prehypertension group, the prevalence of smoker and diabetes mellitus and the level of LDL-C,HDL-C, TC, TG FBG, Scr anduric acid (UA) were higher than that in the ideal blood pressure group (P<0.05). But, the proportions of female were lower in subjects with prehypertension (P<0.05). In the male and female subgroup, the prevalence of smoker and the level of LDL-C, HDL-C, TC, Scr and UA were higher than that in the ideal blood pressure group (Table 2).

**Table 1:** Baseline Characteristics of the Kailuan Community Population.

	whole population(32,385)	ideal blood pressure(12,351)	Prehypertension(20,034)
Age (years)	46.40±11.57	43.91±11.42	47.92±11.4**
Gender: F (%)	8,811(27.2%)	4,485(36.3%)	4,326(21.6%)*
SBP(mmHg)	116.79±11.15	106.77±8.21	122.98±7.61**
DBP(mmHg)	76.24±6.88	70.02±5.79	80.08±4.08**
TG (mmol/L)	1.51±1.23	1.31±1.03	1.62±1.32**
TC (mmol/L)	4.86±1.06	4.79±0.97	4.91±1.11**
LDL (mmol/L)	2.26±0.82	2.23±0.77	2.29±0.85**
HDL (mmol/L)	1.53±0.38	1.52±0.36	1.54±0.39**
FBG (mmol/L)	5.26±1.35	5.13±1.23	5.33±1.42**
UA (μmol/L)	281.83±79.84	275.51±78.72	285.62±80.28*
CR (μmol/L)	85.98±18.12	83.71±16.78	87.35±18.75**
BMI (kg/m^2^)	24.37±3.32	23.62±3.20	24.83±3.20**
Smoking n(%)	11,620(35.9%)	4,310(34.9%)	7,310(36.6%)*
Drinking n (%)	13,434(41.6%)	5,129(41.6%)	8,305(41.5%)
Diabetes n (%)	581(1.8%)	191(1.5%)	390(1.9%)**

Qualitative data are expressed as n (%), quantitative data are mean±SD. SBP, systolic blood pressure; DBP, diastolic blood pressure; TC, total cholesterol; TG, triglyceride; HDL-C, high-density lipoprotein cholesterol; LDL-C: low-density lipoprotein cholesterol; FBG, fasting blood glucose; BMI, body mass index.

*P<0.05,

**P<0.01 compared with the ideal blood pressure group.

**Table 2:** Baseline Characteristics in different gender.

	Male(23574)		Female (8811)	
	Ideal blood pressure(7866)	Prehypertension(15708)	Ideal blood pressure(4485)	Prehypertension(4326)
Age(year)	44.94±11.90	48.08±11.72**	42.24±10.32	47.34±10.16**
SBP (mmHg)	107.74±7.89	123.33±7.53**	105.06±8.46	121.69±7.76**
DBP (mmHg)	70.51±5.79	80.27±4.09**	69.14±5.72	79.42±3.97**
TG (mmol/L)	1.41±1.11	1.68±1.37**	1.15±0.84	1.42±1.08**
TC (mmol/L)	4.81±1.00	4.90±1.13**	4.75±0.92	4.95±1.05**
LDL (mmol/L)	2.29±0.77	2.33±0.83**	2.12±0.76	2.14±0.87
HDL (mmol/L)	1.49±0.36	1.52±0.39**	1.56±0.35	1.58±0.38**
FBG (mmol/L)	5.22±1.31	5.37±1.43**	4.99±1.05	5.19±1.35**
UA (μmol/L)	299.05±76.7	299.01±78.72	234.2±63.81	237.03±65.84*
CR(μmol/L)	88.12±17.02	90.58±18.5**	75.96±13.16	75.63±14.49
BMI(kg/m2)	23.92±3.15	24.91±3.21**	23.09±3.21	24.53±3.62**
Smoking n(%)	4239(54.0%)	7263(46.3%)**	71(1.6%)	47(1.1%)*
Drinking n(%)	4635(59.1%)	8042(51.3%)**	494(11%)	263(6.1%)**
Diabetes n(%)	133 (1.7%)	298 (1.9%)	58(1.3%)	92(2.1%)**

Qualitative data are expressed as n (%), quantitative data are mean±SD. SBP, systolic blood pressure; DBP, diastolic blood pressure; TC, total cholesterol; TG, triglyceride; HDL-C, high-density lipoprotein cholesterol; LDL-C: low-density lipoprotein cholesterol; FBG, fasting blood glucose; BMI, body mass index.

*P<0.05,

**P<0.01 compared with the ideal blood pressure group.

### Follow-up and the occurrence of new CKD events

**Fig 1:**
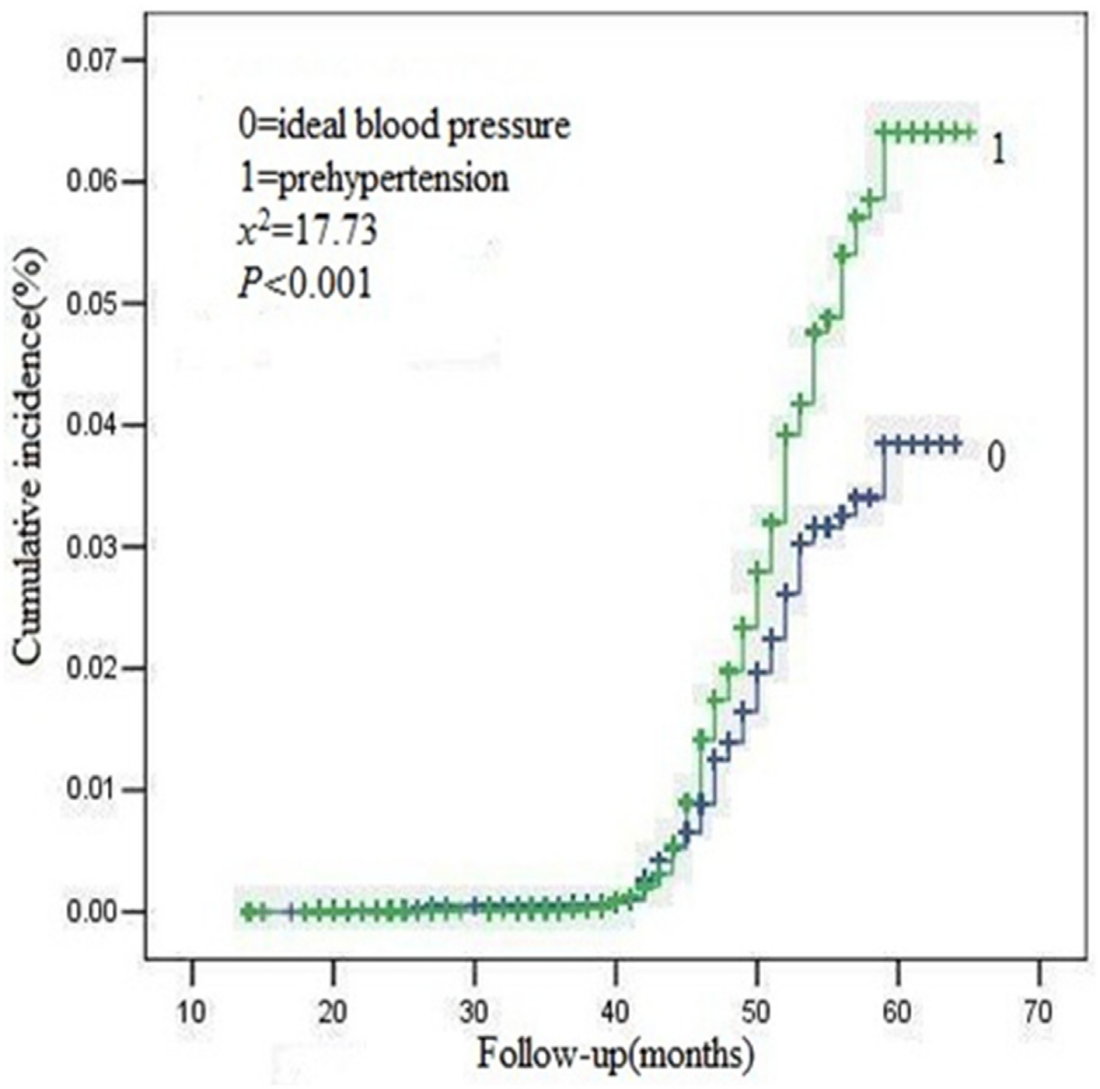
Kaplan-Meier curves for the cumulative new onset CKD events for prehypertension and ideal blood pressure group.

**Fig 2:**
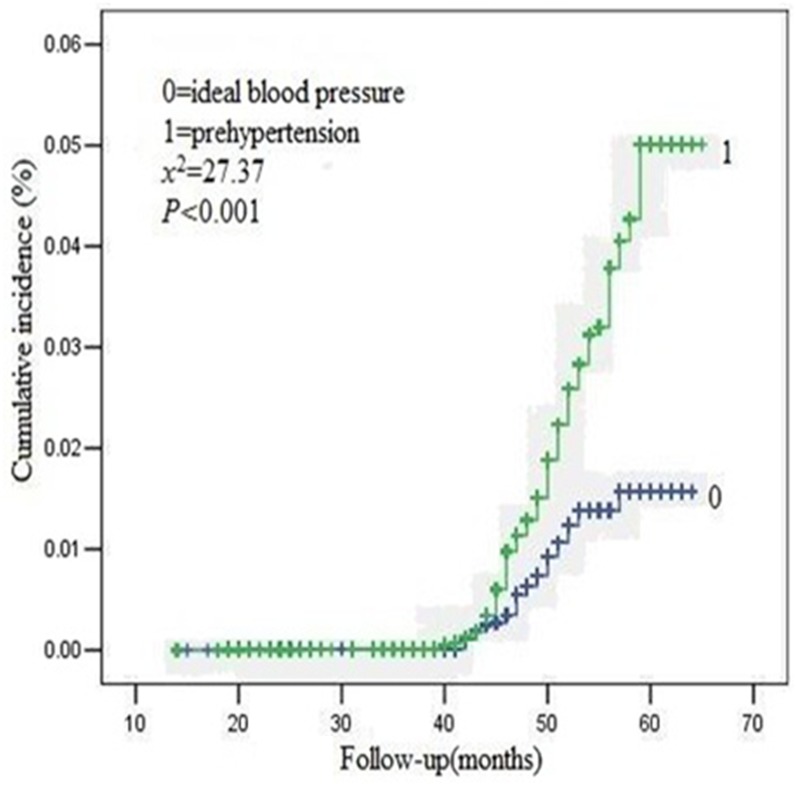
Kaplan-Meier curves for the cumulative new onset CKD events for prehypertension and ideal blood pressure group(male).

**Fig 3:**
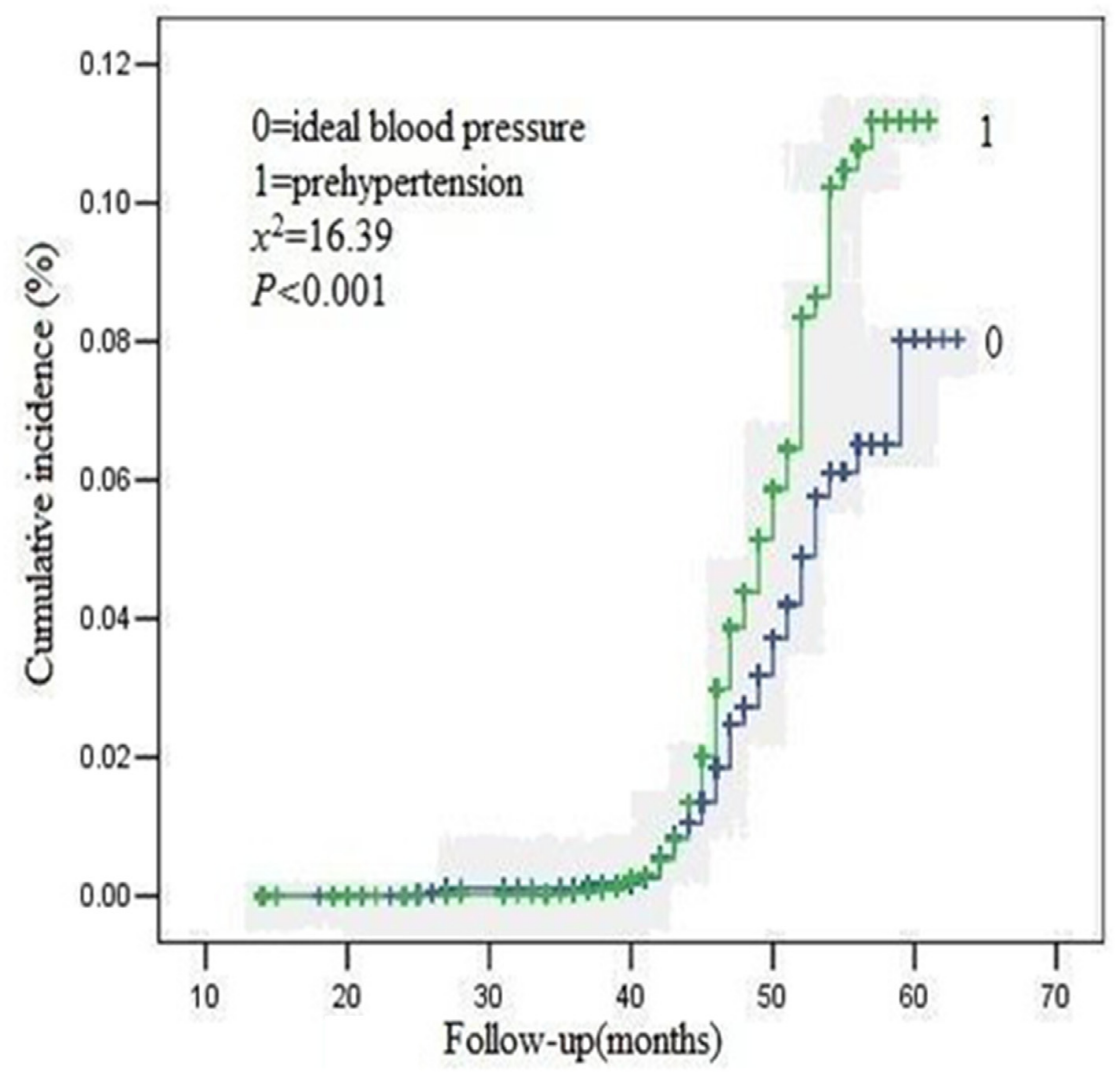
Kaplan-Meier curves for the cumulative new onset CKD events for prehypertension and ideal blood pressure group(female).

The overall median follow-up time was 47 (interquartile range 44–51) months. The Kaplan-Meier survival curve for the occurrence of new CKD events was shown in Figs 1, 2 and 3. The cumulative incidence of new onset CKD events was significantly higher in the prehypertension group than that in the ideal blood pressure group (2.10% vs 1.46%, logrank test, P = 0.0001). 420 out of 20,034 participants with prehypertension and 181 out of 12,351 with ideal blood pressure group occurred CKD events in general population during the follow-up period. The difference in cumulative incidence rates of new CKD events between the two groups was all statistically significant.

Similarly, 200 out of 4,326 female participants, 220 out of 15,780 male participants with prehypertension and 130 out of 4,485 female participants, 51 out of 7,899 male participants with ideal blood pressure group occurred in general population during the follow-up period. The difference in cumulative incidence rates of new CKD events between the two groups was all statistically significant among different gender participants, respectively.

### Association between prehypertension and new onset CKD events

Multivariate Cox hazard regression analysis for the association between prehypertension and predictors of new onset CKD events at follow-up was shown in Table 2. After adjusted for age, sex, history of diabetes mellitus, hyperlipidemia, BMI, TC, LDL-C, TG, UA and FBG, the relative risks (RRs) of new onset CKD events for the prehypertension group were 1.69 (95% CI: 1.41~2.04, P = 0.001) compared with the ideal blood pressure group. Similarly, the RRs of new onset CKD events for the prehypertension group were 1.68 (95% CI: 1.33~2.13 P = 0.001) in females and 2.14 (95% CI: 1.58~2.91 P = 0.001) in males, by adjustment for traditional CV risk factors (Table 3).

**Table 3:** Effect of prehypertension on CKD.

	Model 1*		Model 2*		Model 3*	
	HR95%CI	P	HR95%CI	P	HR95%CI	P
Whole population	1.451.22–1.73	0.001	1.871.56–2.24	0.001	1.691.41–2.04	0.001
Female	1.571.26–1.96	0.001	1.891.5–2.38	0.001	1.681.33–2.13	0.001
Male	2.211.63–2.99	0.001	2.141.58–2.91	0.001	2.141.58–2.91	0.001

*Model 1: Single-factor analysis; Model 2: adjustment for gender and age; Model 3: further adjustment for TG, LDL-C, HDL-C, FBG、and history of smoking, drinking and diabetes based on Model 2.

## Discussion

This is the first prospective study to investigate the relations between prehypertension and the risk of long-term new onset CKD events among the general population in China. We found that the cumulative incidence rates for the occurrence of CKD events were higher (2.10% vs. 1.46%) in participants with prehypertension than that with ideal blood pressure during the four year follow-up. Moreover, further analyses showed that prehypertension was an independent risk factor for new CKD events in the Chinese population.

It is well established that hypertension is a major leading cause of CKD[2, 17]. The prevalence of CKD also increased with BP level stratification, such as normal BP, prehypertension and hypertension[18]. However, up to now, the effect of prehypertension on CKD has not been established. Over the past few years, some cross-sectional epidemiologic studies and Meta-analysis have linked prehypertension to increased risk of CKD[12, 18–20]. However, it is difficult to clear the causal relationship of prehypertension and renal function in cross-sectional studies. Because CKD also leads to BP increased. In our prospective study, we also found that the association between prehypertension and CKD existed. Consistent studies have reported the association of prehypertension and the increased risk for CKD and the progress of ESRD during follow-up[21], whereas other prospective studies of prehypertension and CKD have indicated conflicting results[22, 23]. Several reasons might explain the different results. Firstly, the prevalence of CKD is different in different ethnic populations, which may yield equivocal results regarding the effect of prehypertension on CKD. Secondly, major adverse events are different from our study. CKD(eGFR of less than 60 mL/min per 1.73 m^2^) was defined as a major adverse event in our study. However, ESRD and CKD-related Death, CKD stage 3–5 are defined as major adverse events in Munkhaugen J and Tohidi M studies during follow-up. CKD belongs to subclinical target organ damage, which is preclinical kidney disease, whereas ESRD, CKD-related Death and CKD stage 3–5 belongs to clinical end stage of kidney disease. The severity of major adverse events is different in the different studies, which might explain partially the different results.

The mechanism regarding the impact of prehypertension on CKD remains unclear. Previous study showed that prehypertension was associated significantly with renal arteriosclerosis by kidney biopsy, which may represent a potential pathophysiologic mechanism[24]. Moreover, BP-related progression of renal arteriosclerosis, glomerular sclerosis, renal arteriolar hyalinosis were found, which may lead to renal perfusion and renal ischemia[25], thereby subsequently contributing to decrease in renal function. All these findings may partly explain the effect of prehypertension on CKD.

### Strengths/Limitations

The strength of this study was the first prospective and large sample size study. However, some limitations must be considered. Firstly, our study population included more males than females. However, sex distribution of our study was comparable with that of the whole population of employees. We also found the similar association between prehypertension and CKD in the male and female population respectively. Secondly, the diagnosis of prehypertension for the population was the first time. The duration of prehypertension was not clear. The duration of hypertension was not adjusted in multivariable analysis, which may affect the statistical power. Finally, the diagnosis CKD was based on the creatinine value at a single occasion. However, the diagnosis CKD is consistent with previous published studies that have shown the positive association between prehypertension and CKD[18, 21].

## Conclusion

Prehypertension is an independent risk factor for the occurrence of new CKD events. Therefore, earlier lifestyle interventions for prehypertension may be a strategy for prevention of the occurrence of CKD in the general population.
